# Transcriptome and Metabolite Profiling Reveal Novel Insights into Volatile Heterosis in the Tea Plant (*Camellia Sinensis*)

**DOI:** 10.3390/molecules24183380

**Published:** 2019-09-17

**Authors:** Yucheng Zheng, Pengjie Wang, Xuejin Chen, Yun Sun, Chuan Yue, Naixing Ye

**Affiliations:** College of Horticulture, Key Laboratory of Tea Science, Fujian Agriculture and Forestry University, Fuzhou 350002, Fujian, China; 18094159524@163.com (Y.Z.); 2180311002@fafu.edu.cn (P.W.); 1180311002@fafu.edu.cn (X.C.); sunyun1125@126.com (Y.S.)

**Keywords:** volatile heterosis, *Camellia sinensis*, transcriptome, metabolite

## Abstract

Tea aroma is a key indicator for evaluating tea quality. Although notable success in tea aroma improvement has been achieved with heterosis breeding technology, the molecular basis underlying heterosis remains largely unexplored. Thus, the present report studies the tea plant volatile heterosis using a high-throughput next-generation RNA-seq strategy and gas chromatography–mass spectrometry. Phenotypically, we found higher terpenoid volatile and green leaf volatile contents by gas chromatography–mass spectrometry in the F1 hybrids than in their parental lines. Volatile heterosis was obvious in both F1 hybrids. At the molecular level, the comparative transcriptomics analysis revealed that approximately 41% (9027 of 21,995) of the genes showed non-additive expression, whereas only 7.83% (1723 of 21,995) showed additive expression. Among the non-additive genes, 42.1% showed high parental dominance and 17.6% showed over-dominance. Among different expression genes with high parental dominance and over-dominance expression patterns, KEGG and GO analyses found that plant hormone signal transduction, tea plant physiological process related pathways and most pathways associated with tea tree volatiles were enriched. In addition, we identified multiple genes (CsDXS, CsAATC2, CsSPLA2, etc.) and transcription factors (CsMYB1, CsbHLH79, CsWRKY40, etc.) that played important roles in tea volatile heterosis. Based on transcriptome and metabolite profiling, we conclude that non-additive action plays a major role in tea volatile heterosis. Genes and transcription factors involved in tea volatiles showing over-dominance expression patterns can be considered candidate genes and provide novel clues for breeding high-volatile tea varieties.

## 1. Introduction

Heterosis, or hybrid vigor, which is widely used in agricultural production practice, refers to the phenomenon in which a hybrid progeny has a greater biomass and higher yield and quality than the parents [1]. Since the 20th century, the heterosis breeding technology has been widely applied in cucumber, tomato, corn and other horticultural crops due to the merits shown by the F1 generation [2]. A convincing example is that successful utilization of heterosis has dramatically increased corn production over the last century [3]. To date, three competing but non-mutually exclusive genetic models have been invoked to explain heterosis in both plants and animals, including epistasis [4], overdominance [5] and dominance [6]. However, these classic hypotheses do not fully interpret the exceptional performance of hybrid offspring.

The popularity of sequencing technology has driven an unprecedented development of omics [7]. Attempts by breeders and scientists to expound on the genetic mechanism of heterosis at the molecular level show promise [8,9]. In the super hybrid rice WFYT025, some key genes showing overdominant effects were mapped near QTLs involved with grain number, which might lead to the grain number heterosis [10]. In *Nicotiana tabacum L.*, the key genes involved in nicotine anabolism and transport in the hybrid demonstrated an over-dominant performance that might be the main contributor to nicotine content heterosis [11]. Furthermore, recent research found that the d differentially expressed genes (DEGs) in plant hormone metabolic pathways with up- or down-regulated expression promoted the generation of hybrid vigor. For example, in *Arabidopsis thaliana*, upregulated expression of the transcription factor *PHYTOCHROME-INTERACTING FACTOR (PIF4)* targets the auxin biosynthesis gene *YUCCA8* and the auxin signaling gene *IAA29* may contribute to biomass heterosis [12]. Lower endogenous SA concentrations and down-regulated target gene responses has been found in larger hybrid progeny compared to those of their parental lines [13].

Tea aroma is an important end-user quality parameter of tea products as well as a key indicator for evaluation of tea quality [14,15]. Among these compounds, terpenoid volatiles (TVs) and green leaf volatiles (GLVs) are considered the most critical volatiles for tea aroma, since their content is typically high and thresholds are relatively low [16,17]. In plants, all TVs are derived from two common precursors—isopentenyl diphosphate (IPP) and dimethylallyl pyrophosphate (DMAPP)—that are synthesized by two independent pathways, the cytoplasm mevalonate (MVA) and plastidial methylerythritol phosphate (MEP) pathways [18]. *1-Deoxy-D-xylulose-5-phosphate synthase* (*DXS* gene) and *acetyl-Co A C-acetyltransferase* (*AACT* gene) are the first key committed enzymes in the MEP and MVA pathways, respectively, and they play important roles in TV biosynthesis [19,20]. Subsequently, the generation of TVs is catalyzed sequentially by a series of enzymes. GLVs are biosynthesized from linoleic and linolenic acids through the lipoxygenase (LOX) pathway [21]. In the LOX pathway, some important genes involved in this reaction include SPLA2, which is the first committed enzyme.

Tea is known worldwide for its unique flavor and health benefits [22]. Heterosis breeding technology has achieved notable success in tea breeding. Both the tea cultivars *Camellia sinensis cv*. Jinguanyin (JGY) and *Camellia sinensis cv*. Huangguanyin (HGY) are successful examples of the use of heterosis, because their processed tea products have richer floral and fruity flavors than their parental lines. However, little is known about the molecular mechanisms of aroma heterosis in the tea plant. In the present study, we found that the volatile hybrids content was considerably higher than those of the parents by using TD-GC-MS. Comparative transcriptomics analyses of leaves from the two F1 hybrids and their parental lines were conducted to explore potential molecular mechanisms. In particular, function enrichment analysis was performed for a series of genes that were expressed at higher levels in the offspring than in their parents, and we discover multiple genes associated with aroma heterosis. Our research provides comprehensive and novel insights into the formation of aroma heterosis and new ideas for tea tree hybrid breeding. 

## 2. Results

### 2.1. The Volatile Content was Significantly Increased in Tea Leaves from the F1 Hybrids

The green leaf volatiles monoterpene and sesquiterpene contribute the most to the aroma of tea. We measured green leaf volatiles and terpenes in fresh tea leaves by GC-MS. As shown in Table 1, significant differences were found in the volatile contents among the four different varieties, and obvious aroma heterosis was observed in both F1 generation plants. The total GLV and the mono- and sesquiterpene contents in both F1 generation plants were higher than those of their parental lines. In particular, the GLVs and monoterpene in JGY and mono- and sesquiterpene in HGY were significantly higher than those of their parental lines. We observed that most types of GLVs and monoterpene in the JGY strain, such as hexanal, (*Z*)-2-hexen-1-ol, linalool and geraniol, were significantly higher than the contents in the parent. Similarly, most types of sesquiterpenes, such as germacrene D, and cubenene, were present at significantly higher levels than those in the parent. In addition, the mid-parent heterosis value (MPV) and Over high-parent heterosis value (OPV) were calculated to better understand the degree of heterosis in both F1 generation plants. As shown in Table 2, the sesquiterpene content MPV of HGY was higher than the OPV (over high-parent heterosis value) and MPV in HGY, but the monoterpene content MPV of JGY was higher than HGY. To summarize, the results indicate that the JGY and HGY hybrids demonstrate significant volatile heterosis. Specifically, although both F1 generation tea plants showed volatile heterosis, differences existed between the volatile compound proportions of the two tea varieties.

### 2.2. Higher Gene Expression Similarities were Observed Between the Two F1 Hybrids Than with the Parental Lines 

A cDNA library was constructed with the RNA extracted from the young leaf (one bud and two leaves) samples from the four tea plant varieties (HD, HGY, JGY and TGY). In general, three replicate transcriptome sequences were generated for each tea variety, and a total of 280,000,808 bp raw reads were generated from HD, HGY, JGY and TGY. A total of 272,617,358 clean reads were obtained without adapters after filtering low-quality and N-containing sequences. A total of 40.98 Gb of clean bases was generated from these four tea plants with an error rate of 0.01%, and the average Q20 and Q30 values of those samples were 98.98% and 97.03%, respectively. The average GC content proportion of those samples was 45.49% (Table 3). In addition, we generated 41, 781 genes (one with a FPKM > 0), including 11, 288 novel genes and 30,493 known genes, using the Cufflinks and Cuffmerge software.

**Figure 1:**
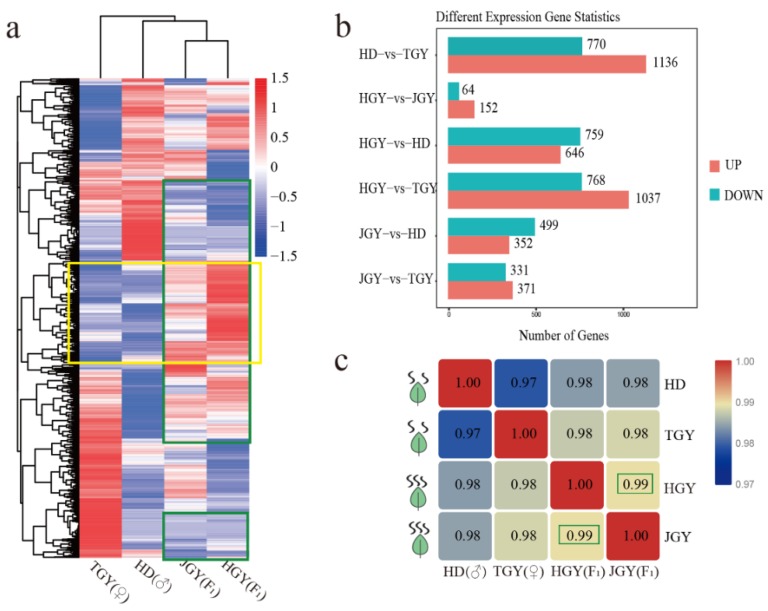
Comparative analysis of the expressed genes detected in two F1 hybrid and parental lines. (**a**) Heat map of DEGs from four different tea cultivar. Clustering by relative expression level value log2 (ratios) of differential genes. Similarly expressed genes between two F1 hybrids were boxed by green and higher expressed genes than parental were boxed by yellow. Red indicates high expression and green indicates low expression (**b**). Differentially expressed genes between different tea varieties at level of *p* > 0.05 and fold change ≥2. (**c**). Correlation matrix of two hybrids and their parents calculated by all the detected genes, two F1 hybrids correlation coefficient were boxed.

To investigate differences in gene expression patterns among the F1 generation and parental lines, 9015 differentially expressed genes (DEGs) were subjected to hierarchical clustering analysis of transcript abundances using the FPKM values. The heat map of the DEGs (Figure 1a) indicated that JGY and HGY had more similar transcriptome profiles than TGY and HD. Some genes with higher expression than those of the parental lines were observed in JGY and HGY. To further discover the heterosis and differences between each tea plant at the transcriptome level, we conducted a comparative analysis between the F1 hybrids and HD or TGY, between the two parents and between the two F1 hybrids at a level of *p* > 0.05 and fold change ≥2. 

As shown in Figure 1b, the least DEGs (64 down- and 152 up-regulated genes) were observed between the two F1 hybrids, which may be due to the same genetic background. Moreover, the correlations among all of the expressed genes proved that the two hybrids achieved the highest correlation coefficient (R = 0.99). These results indicated that the two F1 hybrids had similar gene expression patterns (Figure 1c).

### 2.3. Expression Level Dominance in the Tea Leaf Transcriptome

**Figure 2:**
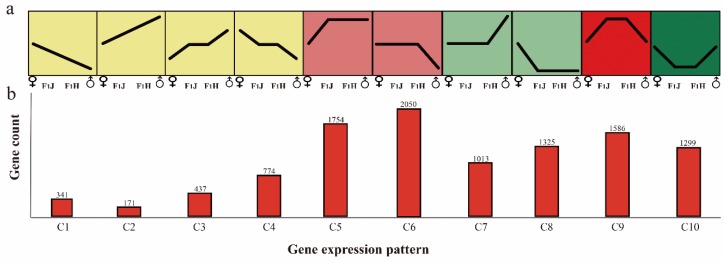
Ten gene expression patterns involved in additive or non-additive expression. (**a**) Expression patterns of 10 types of genes. “♂” means male parent; “♀” means female parent; “F1J” means JGY; “F1H” means HGY. (**b**) The gene number of each pattern. “C” means gene cluster.

To understand the heterosis phenomenon in tea plants comprehensively at the molecular level, a total of 41, 781 genes in the F1 hybrids and parental lines were subjected to trend analysis. As described previously (Materials and Methods) and shown in Figure 2, C1–C4 showed additive expression (~4.12%, 1723 of 41,781), whereas C5-C10 showed non-additive expression (~21.6%, 9027 of 41,781). In particular, C5-C6 showed higher-parental dominance (~9.1%, 3804 of 41,781), whereas C7 and C8 showed lower-parental dominance (~5.6%, 2338 of 41,781); additionally, C9 showed up-regulated overdominance (~3.8%, 1586 of 41,781), whereas C10 showed down-regulated overdominance (~3.1%, 1299 of 41,781). In these 10 expression patterns, the higher-parent dominance pattern (C5–C6) had the highest proportion. In total, hybridization activates most genes in the hybrid progeny, and thus these genes show higher-parent dominance or overdominance. Specific information was listed in Table S2.

**Figure 3:**
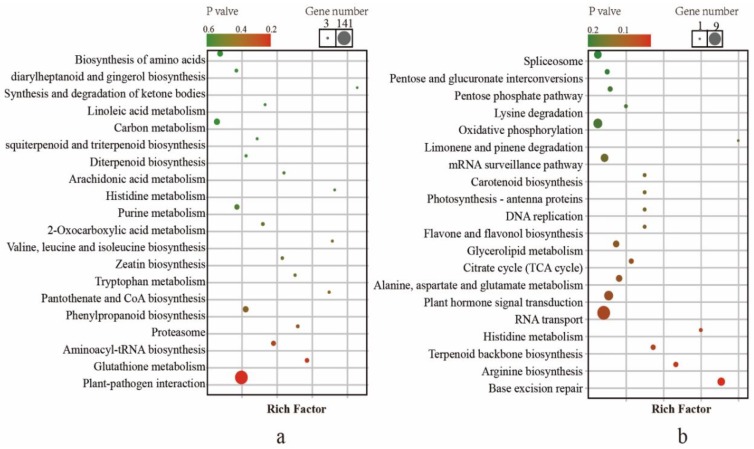
KEGG pathway enrichment for differential gene expression among F1 hybrids and its parents. (**a**) KEGG analysis of gene with a higher-parent dominance pattern. (**b**) KEGG analysis of gene with an over-parent dominance pattern.

To investigate the functions of the genes with a higher-parent dominance pattern and up-regulated overdominance, KEGG and GO enrichment analyses were conducted. The genes involved in higher-parent dominance were enriched (Figure 3a and Table S7). The results indicated that the DEGs were predominantly enriched in “plant-pathogen interaction” and “glutathione metabolism”. In addition, some KEGG pathways involved in plant volatile were enriched, such as “linoleic acid metabolism” and “sesquiterpenoid and triterpenoid biosynthesis”. The genes involved in up-regulated overdominance were also enriched (Figure 3b and Table S8). The enriched genes in the KEGG pathway were involved in terpenoid backbone biosynthesis. Some KEGG pathways involved in plant energy metabolism, such as “pentose and glucuronate interconversions”, “pentose phosphate pathway” and “citrate cycle (TCA cycle)”, were enriched. In particular, the “plant hormone signal transduction” pathway was enriched.

Further analysis of DEGs with a higher-parent dominance pattern indicated that these GO terms were mainly involved in morphological development (trichome morphogenesis, GO:0010090; plant epidermis morphogenesis, GO:0090626 and cell morphogenesis involved in differentiation, GO:0000904), phosphorus and nitrogen metabolic processes (phosphorus metabolic process, GO:0006793; nitrogen metabolic process, GO:1901566; and asparagine biosynthetic process, GO:0006529) and tea plant defense response (defense response, GO:0006952; defense response to bacterium, GO:0009617; and response to stimulus, GO:0050896). The GO terms of DEGs with up-regulated overdominance mainly were involved in volatile biosynthesis, including the isoprenoid biosynthetic process (GO:0008299), terpenoid biosynthetic process (GO:0016114) and terpenoid metabolic process (GO:0006721) (Table 4).

### 2.4. Analysis of Pathways Related to Tea Aroma

**Figure 4:**
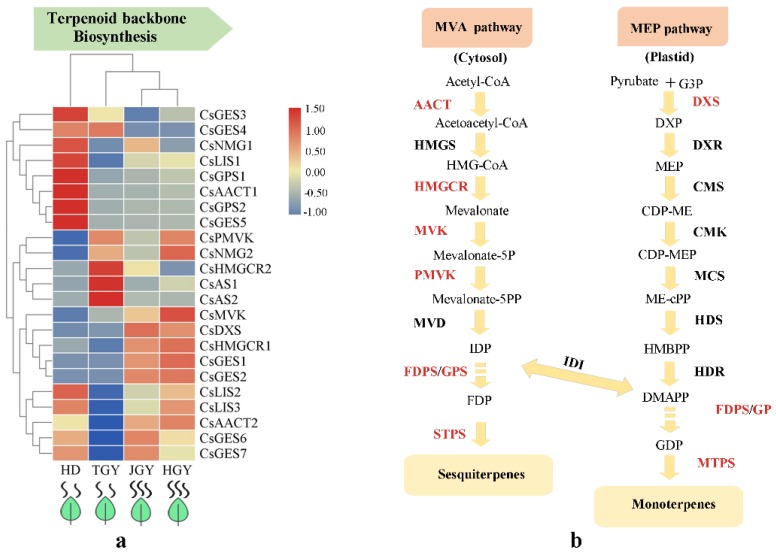
Expression profiles of genes related to monoterpene and sesquiterpene biosynthesis. (**a**) Heat map of the expression pattern of related DEGs in four different tea variety. TBtools software was used for drawing heatmap and the function “scale” was used for normalized the data by rows (**b**) A simplified model of terpenoids pathway in tea plant.

Terpenoid volatiles, C_6_ aldehydes, alcohols, and their esters have a great contribution to the aroma of tea trees. To deeply investigate the mechanisms that regulate the biosynthesis of terpenoid volatiles, C_6_ aldehydes, alcohols and their esters in tea plants, we amply studied the core genes in the pathways and determined quantitative changes in aroma ingredients. In total, 21 DEGs involved in the terpenoid backbone biosynthesis pathway were annotated to encode nine structural genes, including *acetyl-CoA C-acetyltransferase* (*AACT*, 2), *3-hydroxy-3-methylglutaryl coenzyme A reductase* (*HMGCR*, 2), *mevalonate kinase* (*MVK*, 1) *5-phosphomevalonate kinase* (*PMVK*, 1), *geranyl pyrophosphate synthase* (*GPS*, 2) *1-deoxy-D-xylulose 5-phosphate synthase* (*DXS*, 1), *linalool synthase* (*LIS*, 3), *germacrene D synthase* (*GES*, 2) and *β-amyrin synthase* (*AS*, 2). Among these genes, *GES3, GES4, NMG1, LIS1, GPS1, AACT1, GPS2*, and *GES5* had the highest expression in HD. Similarly, *HMGCR, AS1*, and *AS2* had the highest expression in TGY. Notably, *MVK, DXS, HMGCR1, GES1, GES2*, and *AACT2* had higher expression in both HGY and JGY than in HD and TGY (Figure 4 and Table S5). The results indicated that the genes with higher expression in both HGY and JGY might contribute to the higher terpenoid accumulation in HGY and JGY.

**Figure 5:**
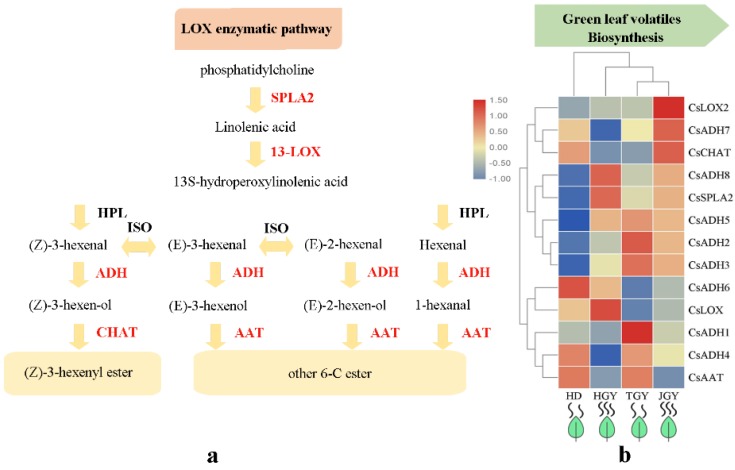
Expression profiles of genes related to green leaf volatiles biosynthesis. (**a**) Heat map of the expression pattern of related DEGs in four different tea variety. TBtools software was used for drawing heatmap and the function “scale” was used for normalized the data by rows (**b**) A simplified model of LOX enzymatic pathway in tea plant.

In addition, we examined the DEGs that mapped to the LOX enzymatic pathway (Figure 5 and Table S6). A total of 13 DEGs were mapped to the LOX pathway, including *alcohol dehydrogenase* (*ADH*, 8), *13-lipoxygenase* (*13-LOX*, 2), *(Z)-3-hexen-1-ol acetyltransferase* (*CHAT*, 1), *alcohol acetyltransferase* (*AAT*, 1) and *secretory phospholipase A2* (*SPLA2*, 1). Among these DEGs, *CsLOX2, CsADH7*, and *CsCHAT* showed the highest expression in JGY. *CsADH8*, *CsSPLA2*, and *CsLOX* showed the highest expression in HGY*. CsADH6* and *CsADH2*, *CsADH3*, and *CsADH1* had the highest expression in HD and TGY, respectively. Further observation found that the DEGs *CsADH8* and *CsSPL2* had higher expression in both TGY and JGY than in HD and TGY. The results indicated that the high *CsADH8* and *CsSPL2* expression might contribute to the accumulation of C_6_ aldehydes, alcohols and their esters in HGY and JGY.

### 2.5. Identification of TFs Involved in the Regulation of Tea Plant Aroma

**Figure 6:**
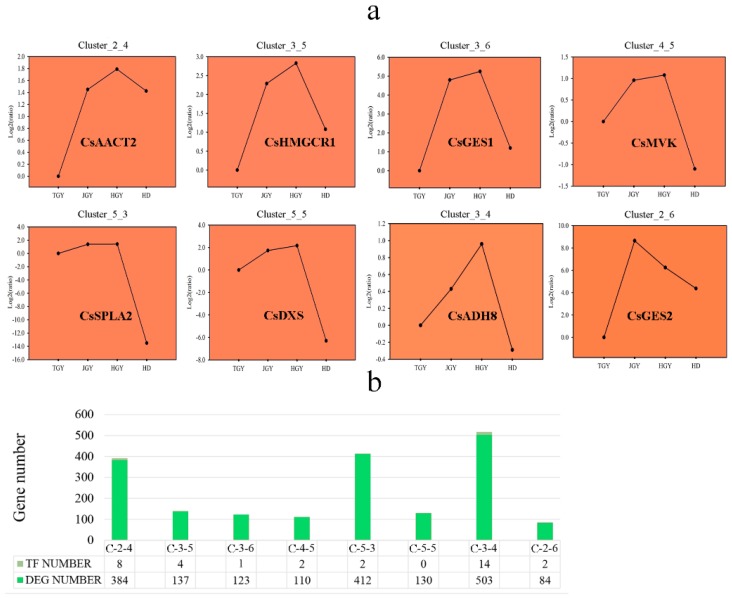
Eight genes involved in volatile biosynthesis were analyzed by SOM (Self-Organizing Feature Map) cluster analysis with R package kohonen. (**a**) Line chart of different expression patterns cluster analysis. (**b**) Number of genes and TFs in different expression patterns.

Transcription factors play important roles in the regulation of volatile biosynthesis in plants. To further probe the reasons for the high volatile content in the F1 generation tea plants, we used SOM cluster analysis to screen out genes with similar expression patterns using genes that had higher expression levels in the F1 generation. As shown in Figure 6a, eight genes were clustered into eight different expression modules. In module Cluster_3_4, *CsADH8* was located with the highest number of genes (503 genes). Among the 503 genes, 14 transcription factors (TFs) were identified using the iTAK (http://bioinfo.bti.cornell.edu/cgi-bin/itak/index.cgi) package. We also obtained some transcription factors with similar expression patterns in other modules. However, no TFs with the same expression patterns were identified in the module in which *CsDXS* was located (Figure 6b).

To further evaluate correlations among the genes involved in volatile biosynthesis and the TFs identified by SOM (Supplementary File 3: Table S3), matrix correlation analysis was conducted with the SPSS software using Pearson’s correlation analysis (Supplementary File 4: Table S4).

**Figure 7:**
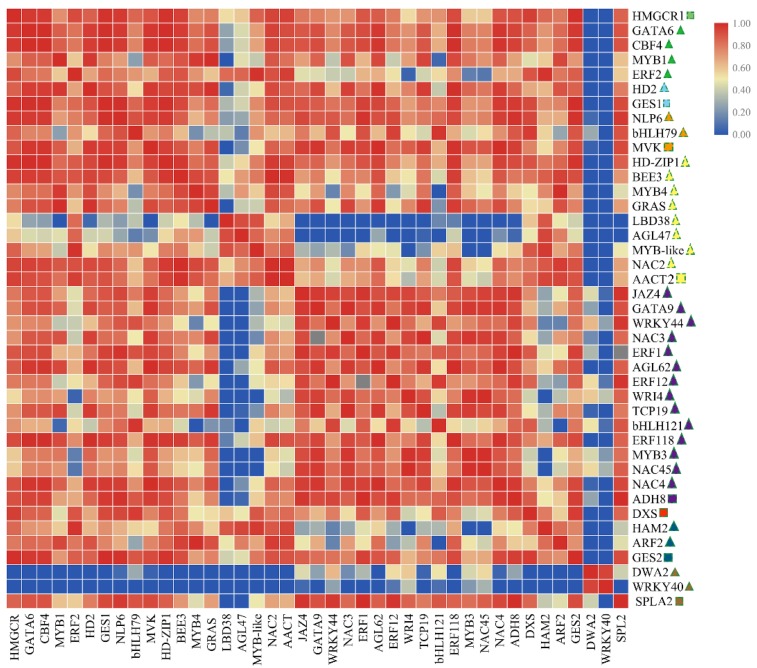
The matrix correlation of genes and TFs involved in volatile biosynthesis (these genes and TFs expression in F1 generation are higher than parental lines). The heatmap drawing was conducted by correlation between genes analyzed by the Spearman test using SPSS 17.0 software and visualized by the TBtools software. Square indicates structural gene; Triangular indicates transcription factors; The same color indicates the same expression pattern in four tea tree.

As shown in Figure 7 and Table S4, the DEGs involved in volatile biosynthesis (*CsAACT2, CsGES1, CsMVK, CsHMGCR1, CsSPLA2, CsADH8* and *CsGES2*) were positively correlated with TFs (*CsMYB1, CsMYB3* and *CsMYB4*, *CsbHLH79, CsbHLH121*, etc.). Meanwhile, we observed a strong positive correlation between transcription factors *(MYB1*, *HD2*, etc.), especially transcription factors of the same family, which showed significant correlations (*CsbHLH79/CsbHLH121* and *CsMYB4/CsMYB1*). The results indicated that TFs might participate in the biosynthesis process underlying tea plant volatile. 

### 2.6. Gene Expression Validated by qRT-PCR

**Figure 8:**
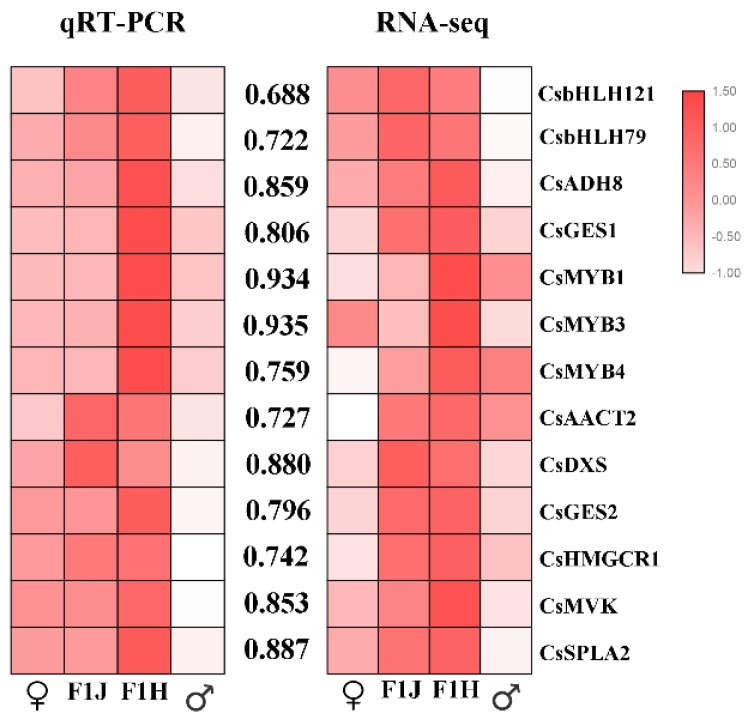
Correlation analysis of candidate genes. The values between the two heatmaps represent correlation value between the expression profiles obtained from RNA-seq and RT-qPCR analysis for each gene. The heatmap drawing was conducted by correlation between genes analyzed by the Spearman test using SPSS 17.0 software and visualized by the TBtools software. “♂”means male parent; “♀” means female parent; “F1J” means JGY; “F1H” means HGY.

To verify the accuracy of the transcriptome data, the transcript abundances of 13 selected DEGs were analyzed by qRT-PCR. In total, five key structural genes in the MVA pathway (*CsAACT2, CsGES1, CsGES2, CsHMGCR1,* and *CsMVK*), one key structural gene in the MEP pathway (*CsDXS*), two key structural genes in the LOX pathway (*CsSPLA2* and *CsADH8*) and some TFs involved in these pathways were selected. Consistent color responses were detected between the qRT-PCR analysis and RNA-seq data (Figure 8). Consistent with the expected results, 13 DEGs were upregulated in the progeny. This result indicated that the transcriptome data and the results of our analysis are reliable.

## 3. Discussion

Heterosis is widely used in cash crops. In tobacco, positive nicotine heterosis has been found in F1 hybrids [23]. In the present study, two offspring (JGY and HGY), superior for the TV and GLV concentrations, were identified using GC-MS (Table 1). Meanwhile, comparative transcriptome analysis was implemented for the tea F1 hybrid (JGY and HGY) and its two parental lines to study the molecular mechanisms contributing to tea volatile heterosis. Interestingly, we found very similar expression patterns and very few DEGs between offspring (Figure 1). KEGG and GO enrichment analysis found that the genes with non-additive expression in offspring were enriched in some pathways which were associated with tea plant volatiles. This result was in accordance with the volatile content of hybrid compared to the two parental lines. Hence, we can conclude that the genes with non-additive expression in offspring may contribute to volatile heterosis in tea plant.

### 3.1. More Meticulous inter-Gene Collaboration May Contribute to Heterosis

In the present study, the comparative transcriptome analysis confirmed similar global expression patterns and only a small proportion of differentially expressed genes between the hybrids. This result indicates the occurrence of a very similar transcriptional reprogramming event between tea F1 hybrids. Transcriptional reprogramming events appear to be common in the hybrid F1 generation and may play a major role in heterosis. In a tobacco intraspecific hybridization experiment, genes showing a significant higher-parental expression level dominance pattern were the main cause of nicotine heterosis [11]. The same result was also confirmed in *Arabidopsis*, in which overdominance and underdominance lead to heterosis [24,25]. Obviously, alteration in gene expression levels in the F1 hybrid generation is the molecular basis of heterosis [12]. Interestingly, similar expression patterns were observed in the two F1 hybrids in our study. In addition, hybrid progeny gene expression network analysis found that the hybrids possessed an enriched number of partial correlations at the genome-wide level compared to those of their parental lines [26]. These results may indicate that variation in gene expression in the F1 generation with heterosis is not in a random or disordered state but instead represents a series with tighter regulation. More meticulous inter-gene collaboration seems to enable plants to utilize resources more efficiently and thus to produce heterosis. This new perspective suggests that regulatory control of heterosis is achieved through successive cascades of gene activity, which has provided us with a better understanding of the phenomenon [27]. The explanation is based on more complicated epigenetic regulation.

### 3.2. Non-Additive Expression Plays A Dominant Role in The Formation of Tea Heterosis

A discussion of whether additive or non-additive gene expression patterns play a dominant role in the formation of heterosis has been ongoing for a long time. A large proportion of genes show additive expression patterns, which are considered to be the main cause of heterosis in both maize and rice hybrids and their inbred parents [28,29]. Analysis of immature ear tissues of maize also found that additive expression was the main reason for heterosis, whereas non-additive expression was negatively correlated with heterosis [30]. Some researchers believe that non-additive expression hinders the formation of plant heterosis [31]. Moreover, many studies have demonstrated that genes showing dominant or transgressive expression levels confer the F1 generation with a larger biomass and stronger adaptability. In *Drosophila*, a large proportion of genes showing non-additive expression patterns was considered the origin of heterosis [32]. Studies of maize [33], wheat [34], and cotton [35] reached the same conclusion. In this study, ~21.6% (9027 of 41,781) of the genes showed non-additive expression, whereas only ~4.12%, (1723 of 41,781) showed additive expression. Our conclusion is consistent with the latter pattern. Genes with non-additive expression play a key role in the volatile heterosis manifestation in tea plants. Among all of the non-additive genes, 42.1% showed higher-parent dominance and 17.6% showed overdominance. The classical genetic hypothesis indicates that the genomes from the two parents provide their advantageous alleles in the nucleus of the F1 hybrid, resulting in higher-parent dominance and leading to hybrid vigor [6]. The overdominance hypothesis indicates that heterogeneous combination of alleles from both parents generates a performance of “1 + 1 > 2” [5]. However, recent reports have suggested that non-additive expression might be influenced by the contribution of regulators, such as transcription factors (TFs), which seems to better explain the above phenomenon [36]. Indeed, we found that 33 TFs, which were highly correlated with volatile genes, exhibited differential expression in the hybrid compared with either parent. 

Subsequently, we performed KEGG and GO enrichment analyses for the genes with non-additive expression. The results showed that some of the genes were enriched in pathways that were highly correlated with heterosis, such as “plant hormone signal transduction”. Recently, a breeder found that IAA-targeted gene activity was frequently correlated with a greater leaf cell number heterotic phenotype and that SA-targeted gene activity was negatively correlated with heterosis [13]. Increased expression of the transcription factor *PIF4* may contribute to biomass heterosis by targeting the auxin signaling gene IAA29 [12]. Our results demonstrated that upregulated hormone-related genes in tea plants might contribute to basic development of the plants. Additionally, “biosynthesis of amino acids”, “carbon metabolism”, “citrate cycle (TCA cycle)”, and “alanine, aspartate and glutamate metabolism” are related to the basic metabolic activity of plants. Similarly, GO enrichment analysis found that plant energy metabolism GO terms were enriched, such as “phosphorus metabolic process”, “organonitrogen compound biosynthetic process” and “cellular nitrogen compound catabolic process”; plant development processes were also enriched, such as “trichome morphogenesis”, “plant epidermis development”, and “tissue development”. Although these pathways have not been reported to be related to heterosis in previous studies, they are closely related to physiological processes in tea plants and provide the necessary energy sources and structural components for tea plant volatile heterosis. Notably, we found that most pathways associated with tea plant volatiles were enriched, including the downstream pathways “diterpenoid biosynthesis” and “sesquiterpenoid and triterpenoid biosynthesis”, the upstream pathway “terpenoid backbone biosynthesis” and the GLV synthesis pathway “linoleic acid metabolism”. The GO enrichment analysis showed the same results (“terpenoid biosynthetic process”, “lipid biosynthetic process”, “terpenoid metabolic process” and “sesquiterpene synthase activity”). These non-additive genes involved in tea plant volatile biosynthesis may play key roles in tea plant volatile heterosis”.

### 3.3. Genes with Altered Expression Levels May Contribute to Volatile Heterosis

**Figure 9:**
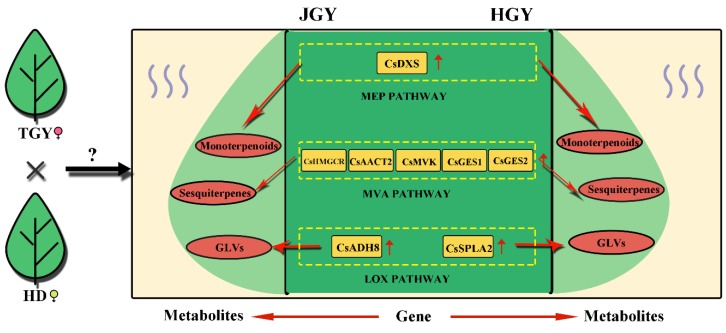
Schematic diagram of key genes and metabolites of terpenoid and green leaf volatiles in F1 generation (HGY and JGY). Yellow square indicates gene with higher expression level in F1 generation than in parental line; Red oval indicates metabolite with higher content in F1 generation than in parental line. Solid line represents direct action.

The MEP and MVA pathways involve a series of consecutive enzymes and are mainly responsible for the synthesis of isopentenyl diphosphate (IPP) and dimethylallyl pyrophosphate (DMAPP), respectively, which are the common precursor substances of terpenoid [18]. *DXS* is the first key rate-limiting enzyme in the MEP pathway [37]. *Arabidopsis* expressing a higher *DXS* level (overexpressors) demonstrates a higher isoprenoid final product level [38], and more than twice the accumulation of various isoprenoid final products was observed in potatoes and tomatoes overexpressing the *DXS* gene [39,40]. *HMGCR* is the first key rate-limiting enzyme in the MVA pathway [41]. Overexpression of *PgHMGR1* enhanced terpenoid and sterol production in *Arabidopsis* and ginseng [42]. An embryo-lethal phenotype has been observed in A. thaliana with an *AACT2* mutation, indicating that *AtAACT2* plays an important role in isoprenoid synthesis [20]. *MVK* is a potential regulatory enzyme of the isoprenoid biosynthetic pathway [43]. In this study, *CsDXS*, *CsHMGR1*, *CsAACT2*, *CsMVK* and the terpene synthase gene (TPS) *CsGES* showed overdominance expression patterns in the tea hybrids. Correlation analysis found that the *CsDXS* expression level was significantly correlated with the total amount of monoterpenes in tea plants (r = 0.978, *p* = 0.022), whereas *CsHMGR1* expression was correlated with total amount of sesquiterpenoids (r = 0.818). Similarly, we observed that *CsADH8* and *CsSPLA2* in the LOX enzymatic pathway were upregulated in the F1 hybrids. *CsADH8* can reduce the aldehyde formed by HPL catalysis to the corresponding alcohol, which directly produces aroma substances [44]. In summary, these results indicate that these eight genes play pivotal roles in the formation of volatile heterosis in both JGY and HGY (Figure 9). 

### 3.4. Transcription Factors (Tfs) Probably Underlie Heterosis

TFs are an important cause of changes in gene expression. Researchers think one plausible molecular mechanism is that hybrids are caused by interactions between transcription factor allelic promoter regions [45]. We conducted SOM cluster analysis to filter out TFs that were correlated with the above eight genes. In this study, we identified 33 TFs that were upregulated in the F1 hybrids and correlated with the above eight genes. Previous studies found that *ptMYB* contributed to terpene synthesis in conifer trees similar to *AtMYC* in *Arabidopsis* [46,47]. *WRKY3* and *WRKY6* were found to be related to the accumulation of volatiles during continuous insect attacks in *Nicotiana attenuate* [48]. Three *CsMYB* (*CsMYB1, CsMYB3* and *CsMYB4*), two *CsMYC (CsbHLH79* and *CsbHLH121*) and two *CsWRKY* (*CsWRKY40* and *CsWRKY44*) genes were identified among the 33 TFs. Furthermore, in addition to the seven reported TFs, the remaining 26 novel TFs may play an important role in volatile heterosis.

## 4. Materials and Methods 

### 4.1. Plant Material Preparation

The parental lines (*Camellia sinensis cv*. Tieguanyin (TGY), female parent and *Camellia sinensis cv*. Huangdan (HD), male parent) and F1 hybrids (*Camellia sinensis cv.* Jinguanyin (JGY) and *Camellia sinensis cv.* Huangguanyin (HGY) were obtained from Ningde Vocational and Technical College (Fujian China, E119°66′ N27°10′) consistent with the tea plant growth period in May 2018. Indeed, all of tea plants were grown under the same cultivation practices. Shoot tips with two unfolded leaves samples were freshly picked as follows: each variety samples was picked from 10 tea trees, respectively, mixed as a biological repetition. A total of 300 g of fresh leaf samples were picked from each tea plant variety for detection of released volatiles and transcriptome analysis (100 g of tea leaves were harvested for each biological replicate; we collected three biological replicates from the four cultivars). All samples were packed with tinfoil, placed into liquid nitrogen and then maintained at −80 °C. 

### 4.2. Thermal Desorption Combined with Gas Chromatography Mass Spectrometry (TD-GC-MS) Analysis of Volatile Compounds in the Leaves

**Figure 10:**
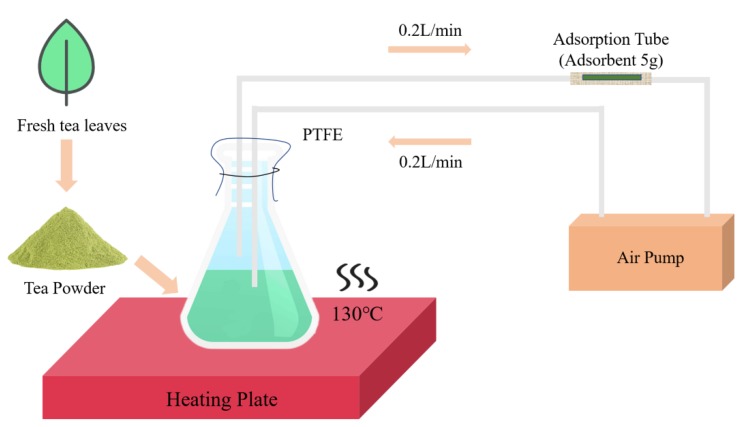
Simplified sampling process.

First, we created a simple and efficient equipment to gather more tea volatiles (Figure 10). A total of 250 g of vacuum freeze-dried leaf samples (total 1000 g) was ground into powder and then filtered with 50 mesh sieves. Then, 5 g of ground tea powder was placed into a beaker with 100 mL of distilled boiling water, and 15 μL of ethyl decanoate (100 ppm) was added as an internal standard. Next, the beaker mouth was quickly sealed with a membrane (polytetrafluoroethylene) connected to an air pump (flow rate 0.2 L/min) through a pipe (polytetrafluoroethylene) with an adsorption tube (5 g of adsorbent). The beaker containing the tea mixture was placed on a heating plate (130 °C) for 30 min and shaken every 5 min. Finally, the enriched adsorption tube was placed on the thermal desorption autosampler.

The Auto TD A thermal desorption system (Colin Corporation, Chengdu, China) was used to gather tea volatiles. Tubes enriched with trapped volatiles from fresh tea leaves were totally desorbed (desorption time: 0.05 min, desorption temperature: 250 °C, flow: 35 mL/min, and split: 25 mL/min). Next, the volatilized component reached the cold trap (25 °C) and was desorbed (desorption temperature: 300 °C, flow: 35 mL/min, and split: 5 mL/min). Finally, the volatiles were carried by carrier gas into the gas chromatograph (transmission temperature: 200 °C, carrier gas pressure: 60.0 kPa, and valve temperature: 200 °C).

The TQ8040 GC-MS (Shimadzu Corporation, Kyoto, Japan) was used for analysis. Helium (99.99%) was used as the carrier gas (pressure: 49.5 kPa, total flow: 9 mL/min, column flow: 1 mL/min, line speed: 36.1 cm/s, purge flow: 3 mL/min, and split ratio: 5). The initial temperature of the column oven was set at 40 °C (3 min), and the inlet temperature was set at 240 °C. The column parameter was 30 m × 0.25 mm × 0.25 um. The MS conditions were as follows: ion source temperature: 230 °C; interface temperature: 280 °C; and total program time: 36 min. Specifically, the GC oven temperature was held at 40 °C for 3 min, increased to 120 °C for 5 min at a rate of 5 °C/min, and finally increased to 240 °C for 8 min at a rate of 30 °C/min. Every volatile compound was identified in the NIST library according to both its retention time and mass spectrum values. We calculated the relative concentration of the target released from the volatile using Wang’s method [49].

### 4.3. Transcriptome Sequencing Preparation and Data Analysis

Total RNA samples (four tea plant varieties with three replicates) were prepared using the pBIzol kit (BIOFLUX, Hangzhou Bori Technology Co., Ltd., Hangzhou, China), and RNA degradation and contamination were monitored on 1% agarose gels. The RNA purity was checked using a Nano Photometer spectrophotometer (IMPLEN, Los Angeles, CA, USA). The RNA concentration was measured using the Qubit RNA Assay Kit in a Qubit 2.0 Fluorometer (Life Technologies, Gaithersburg, MD, USA). RNA integrity was assessed using the RNA Nano 6000 Assay Kit for the Agilent Bioanalyzer 2100 system (Agilent Technologies, Santa Clara, CA, USA). After all samples were tested (RIN > 0.8), each RNA sample (2 μg) was used for library sequencing. The library construction and RNA-seq were performed by the Allwegene Biotechnology Corporation (Beijing, China) according to the manufacturer’s instructions. A total of 12 libraries (four samples with three biological replicates) were sequenced on the Hiseq 4000 platform (Illumina, San Diego, CA, USA). 

### 4.4. Gene and Expression Annotation

The fastq files of the raw reads were filtered using in-house Perl scripts, and adapters and low-quality reads were removed by Trimmomatic (v0.33). Then, the GC contents and the Q20 and Q30 values of the clean data were calculated to ensure the accuracy of the downstream analyses based on these high-quality clean reads. The Trinity method was used for transcriptome assembly. Then, these clean reads were mapped to the tea plant reference genome sequence [50] by TopHat2 with the mismatch set to 2 by default and all other parameters set as the default values. Gene function was annotated based on the following databases: nr (NCBI nonredundant protein sequences); nt (NCBI nonredundant nucleotide sequences); Pfam (Protein family); KOG/COG (Clusters of Orthologous Groups of Proteins); Swiss-Prot (a manually annotated and reviewed protein sequence database); KO (KEGG Ortholog database); and GO (Gene Ontology). The gene expression levels of each sample were analyzed using the HTSeq software with the model set to union. The gene expression levels were measured using FPKM (Fragments per kilobase of exon model per million mapped reads) [51]. Read counts based on the gene expression level analysis were used for the gene differential expression analysis with DESeq (1.10.1). Typically, the standard for differential gene selection is a q-value<0.05. 

### 4.5. Cluster and Trend Analysis

To identify genes with the same expression patterns, all genes were used for the SOM (Self-Organizing Feature Map) cluster analysis with the R package kohonen, which is an unsupervised learning neural network. We performed a trend analysis for all expressed genes. The parameter is |log2 (fold change)| >2 and a q-value <0.05. This trend analysis was performed at http://www.omicshare.com/tools.

### 4.6. Validation of the Accuracy of the Transcriptome Data by Quantitative Real-Time PCR Analysis

To validate the accuracy of the transcriptome data, 13 candidate genes involved in the volatiles synthesis pathways were selected. All specific primers for these genes were designed with the Primer6 software; the specific primer sequences are listed in detail in (Supplementary File 1: Table S1). RNA templates were harvested from four tea plant varieties (Tieguanyin, Huangguanyin, Jinguanyin and Huangdan) using the RNAprep Pure Plant Kit (polysaccharide- and polyphenolic-rich) (Tiangen, Beijing, China). cDNAs were synthesized from the RNA templates using the TransScript® One-Step gDNA Removal and cDNA Synthesis SuperMix (Transgen, Beijing, China). To achieve a higher synthesis efficiency, RNA templates, specific primers and RNase-free water were mixed first, incubated at 65°C for 5 min and then placed on ice for 2 min. Next, the remaining reaction components were added. The qRT-PCR was performed on the CFX96™ real-time PCR system (Bio-Rad, USA) with a 10 μL reaction volume using the TransStart Tip Green qPCR superMix kit (Transgen). GAPDH was selected as the reference gene. All samples were analyzed in three biological and three technical replicates, and the results were calculated using 2^−ΔΔCT^ [52].

### 4.7. Definition of Expression Patterns of Degs Between F1 Hybrids and Parental Lines

According to the previously described method [35], a FPKM value (F1 hybrid) between the two parents of V_parent1_ > V_hybrid1_ > V_hybrid2_ > V_parent2_ or V_parent1_ > V_hybrid1_ = V_hybrid2_ > V_parent2_ was defined as an additive expression mode. An FPKM value (F1 hybrid) close to the higher parent value (V_parent1_ = V_hybrid1_ = V_hybrid2_ > V_parent2_) was defined as a higher-parental dominance expression mode, and a FPKM value (F1 hybrid) close to the lower parent value (V_parent1_ = V_hybrid1_ = V_hybrid2_ < V_parent2_) was defined as a lower-parent dominance expression mode. If the FPKM values of both hybrids were higher than those of the two parents, we defined this expression mode as upregulated overdominance; conversely, the opposite trend was defined as down-regulated overdominance. “>” means statistically higher (*p* < 0.05 and fold change ≥2), “<” means statistically lower (*p* < 0.05 and fold change ≥2) and “=” means statistically similar.

### 4.8. Statistical Analysis

All data were analyzed based on the results of T-tests using SPSS Statistics 17 (SPSS Inc., Chicago, IL, USA). The two sets of data are marked with different letters to indicate statistically significant differences (*p*-value < 0.05). Correlations were analyzed based on the Pearson analysis results. All data are presented as the mean ± standard error (SE, *n* = 3). The Mid-parent heterosis value (MPV) and Over high-parent heterosis value (OPV) was calculated as previous described [10]: MPV (%) = (F1 − MP)/MP × 100, HPV (%) = (F1 − HP)/HP × 100, F1 = performance of F1 generation, HP = performance of high-value parent, MP = mid-parent average [(Parent1 + Parent2)/2].

## 5. Conclusions

In this study, we conducted a comprehensive comparative transcriptional analysis of parental lines and their offspring to investigate possible molecular mechanisms leading to volatile heterosis in the tea plant. We systematically classified the acquired DEGs and found that non-additive expression was the main mode of inheritance. In addition, we found some genes and TFs that were significantly upregulated in pathways associated with tea volatile in the progeny. Overall, these data provide novel insights into the possible molecular mechanisms leading to volatile heterosis in the tea plant, which offers new opportunities for revealing the heterosis of higher volatiles.

## Figures and Tables

**Table 1:** Volatiles content of F1 hybrids and their parents (HD × TGY) (ng/g).

RT	CAS	RI	MS	Compounds	HGY	JGY	TGY	HD
5.78	66-25-1	806	96	Hexanal	56.1 ± 12.86a	91.4 ± 17.77b	43.92 ± 11.85a	42.33 ± 5.75a
7.36	6728-26-3	814	95	(E)-2-Hexenal	72.5 ± 21.88a	225.95 ± 40.01b	127.13 ± 11.51c	60.23 ± 8.07a
7.47	928-97-2	868	96	(*E*)-3-Hexen-1-ol	92.84 ± 30.95b	80.86 ± 7.94b	77.22 ± 3.49b	84.44 ± 22.07a
7.76	928-94-9	868	97	(Z)-2-Hexen-1-ol	10.66 ± 1a	13.78 ± 1.61b	5.83 ± 0.61c	3.28 ± 0.59d
7.89	111-27-3	860	92	1-Hexanol	84.97 ± 14.18a	69.65 ± 8.29a	33.79 ± 12.23b	27.11 ± 9.48b
12.41	3681-71-8	992	96	(Z)-3-Hexen-1-ol acetate	116.31 ± 32.14b	102.98 ± 22.15b	115.78 ± 19.19b	66.79 ± 2.6a
Sum	(GLVs)				432.93 ± 79.04bc	584.62 ± 91.38c	400.67 ± 66.53b	284.18 ± 40.92a
14.73	5989-33-3	1529	92	Linalool oxide 1	289.92 ± 92.76b	284.78 ± 25.62b	115.73 ± 23.83a	190.75 ± 4.42b
15.32	34995-77-2	1529	95	Linalool oxide 2	509.3 ± 98.74a	287.01 ± 20.36a	241.48 ± 69.98a	116.86 ± 59.02a
15.73	78-70-6	1082	94	Linalool	387.86 ± 66.16a	447.12 ± 51.29a	222.01 ± 73.24b	310.64 ± 42.1b
19.42	432-25-7	1204	90	β-Cyclocitral	11.72 ± 2.29a	4.15 ± 0.79b	3.55 ± 1.06b	2.34 ± 0.83b
19.73	106-26-3	1228	90	*cis*-Citral	8.83 ± 3.18b	20.42 ± 2.65a	8.31 ± 0.75b	2.97 ± 0.23c
19.58	106-25-2	1228	92	(*Z*)-Geraniol	4.88 ± 1.22b	6.75 ± 1.07b	7.69 ± 1.71b	32.9 ± 6.66a
20.67	106-24-1	1228	93	Geraniol	288.28 ± 29.56b	505.67 ± 42.21a	164.19 ± 26c	257.69 ± 58.04b
Sum	(monoterpene)				1500.79 ± 396.8a	1555.90 ± 143.99a	766.43 ± 117.94b	916.7 ± 73.39b
24.63	23986-74-5	1515	85	Germacrene D	5.49 ± 1.01a	1.27 ± 0.08b	0.34 ± 0.09b	0.47 ± 0.05b
26.14	15423-57-1	1344	84	Germacrene B	6.14 ± 1.76a	1.47 ± 0.17b	1.39 ± 0.33b	1.02 ± 0.15b
27.14	502-61-4	1458	82	α-Farnesene	0a	7.5 ± 0.64b	0a	0a
27.49	16728-99-7	1344	86	Cubenene	10.21 ± 1.22a	0c	6.4 ± 1.61b	0c
27.68	142-50-7	1564	88	Nerolidol 2	20.74 ± 3.78a	14.74 ± 3.81a	14.15 ± 1.77a	14.69 ± 1.23a
Sum	(sesquiterpene)				42.58 ± 6.77c	24.98 ± 4.19b	22.28 ± 2.58b	16.18 ± 1.18a

The data are shown as mean ± standard deviation of triplicate tests. Significance (*p* < 0.05) is indicated by different letters in the same row. Abbreviations: CAS, Chemical Abstracts Service: RT, retention time; RI, retention index; MS, matching score.

**Table 2:** Heterosis of volatiles content of hybrid combination (HD × TGY) in tea.

F1	Compound	Mid-Parent Heterosis Value	Over High-Parent Heterosis Value
HGY	Green leaf volatiles	26.4%	8.0%
	monoterpene	78.3%	63.7%
	sesquiterpene	121.4%	47.7%
JGY	Green leaf volatiles	70.1%	45.9%
	monoterpene	84.9%	69.7%
	sesquiterpene	65.9%	12.1%

**Table 3:** Sequencing data statistics derived from HD, HGY, JGY and TGY.

Sample	Raw Reads	Clean Reads	Clean Bases	Error (%)	Q20 (%)	Q30 (%)	Gc (%)
HD_1	23334614	22395426	3.36G	0.01%	99.15%	97.36%	45.76%
HD_2	23333820	22402952	3.36G	0.01%	99.10%	97.25%	45.67%
HD_3	23332984	22387946	3.36G	0.01%	97.54%	94.10%	45.83%
HGY_1	23333348	22889486	3.43G	0.01%	99.09%	97.24%	45.39%
HGY_2	23334211	22772393	3.43G	0.01%	99.12%	97.33%	45.37%
HGY_3	23334388	22809487	3.43G	0.01%	99.10%	97.27%	45.57%
JGY_1	23332300	22881487	3.42G	0.01%	99.09%	97.24%	45.25%
JGY_2	23332329	22766832	3.42G	0.01%	99.10%	97.24%	45.97%
JGY_3	23332293	22850808	3.42G	0.01%	99.12%	97.30%	45.34%
TGY_1	23333500	22974617	3.45G	0.01%	99.14%	97.35%	45.03%
TGY_2	23333469	22667735	3.45G	0.01%	99.13%	97.34%	45.48%
TGY_3	23333552	22818189	3.45G	0.01%	99.12%	97.32%	45.25%
Total	280000808	272617358	40.98G				

**Table 4:** GO analysis of DEGs involved in higher parent dominance and up regulated overdominance.

GO ID	GO Term	Gene Ratio	*p*-value	FDR
Higher parent dominance				
GO:0006528	asparagine metabolic process	6	0.000721	0.516173
GO:0006529	asparagine biosynthetic process	6	0.000721	0.516173
GO:0044237	cellular metabolic process	436	0.000934	0.516173
GO:0010026	trichome differentiation	4	0.002543	0.702621
GO:0010090	trichome morphogenesis	4	0.002543	0.702621
GO:0090558	plant epidermis development	7	0.003961	0.74574
GO:0009888	tissue development	13	0.00612	0.74574
GO:0006796	phosphate-containing compound metabolic process	162	0.00683	0.74574
GO:0006793	phosphorus metabolic process	162	0.007318	0.74574
GO:0000904	cell morphogenesis involved in differentiation	5	0.007927	0.74574
GO:0090626	plant epidermis morphogenesis	5	0.007927	0.74574
GO:0050896	response to stimulus	134	0.010821	0.74574
GO:0019538	protein metabolic process	199	0.011409	0.74574
GO:0006952	defense response	30	0.018033	0.74574
GO:0042742	defense response to bacterium	10	0.019967	0.74574
GO:0009690	cytokinin metabolic process	4	0.025745	0.74574
GO:0009617	response to bacterium	10	0.027516	0.74574
GO:0045595	regulation of cell differentiation	3	0.037732	0.74574
GO:1901566	organonitrogen compound biosynthetic process	89	0.039279	0.74574
GO:0030154	cell differentiation	10	0.04827	0.74574
Up regulated overdominance				
GO:0008299	isoprenoid biosynthetic process	8	0.003323	0.603651
GO:0016114	terpenoid biosynthetic process	6	0.010173	0.626311
GO:0051321	meiotic cell cycle	5	0.014234	0.626311
GO:0009658	chloroplast organization	4	0.01434	0.626311
GO:0044786	cell cycle DNA replication	2	0.020165	0.626311
GO:0044270	cellular nitrogen compound catabolic process	6	0.031197	0.626311
GO:0009648	photoperiodism	2	0.038089	0.626311
GO:1903046	meiotic cell cycle process	4	0.040315	0.626311
GO:0008610	lipid biosynthetic process	12	0.040691	0.626311
GO:0006721	terpenoid metabolic process	6	0.041973	0.626311
GO:0034004	germacradienol synthase activity	3	0.01149	0.522775
GO:0052577	germacrene-D synthase activity	3	0.01149	0.522775
GO:0010334	sesquiterpene synthase activity	3	0.01879	0.527904

## Data Availability

Specific transcriptome sequencing results have been uploaded to the Sequence Read Archive database (SRA accession: PRJNA526005).

## Supplementary Materials

Table S1: The primer sequences used for qRT-PCR amplification; Table S2: Number of genes with different expression levels; Table S3: The result of SOM cluster analysis; Table S4: Correlation among the genes involved in volatile biosynthesis and the TFs identified by SOM; Table S5: FPKM value of the genes in the terpenoid backbone biosynthesis pathway; Table S6: FPKM value of the genes in the LOX pathway; Table S7: KEGG enrichment analyses for the genes involved in higher-parent dominance; Table S8: KEGG enrichment analyses for the genes involved in over-dominance.
